# Rational Construction of Pt Incorporated Co_3_O_4_ as High-Performance Electrocatalyst for Hydrogen Evolution Reaction

**DOI:** 10.3390/nano14110898

**Published:** 2024-05-21

**Authors:** Peijia Wang, Yaotian Yan, Bin Qin, Xiaohang Zheng, Wei Cai, Junlei Qi

**Affiliations:** 1 School of Materials Science and Engineering, Harbin Institute of Technology, Harbin 150001, China; 17826856854@163.com (P.W.);; 2 Key Laboratory of Magnetic Molecules and Magnetic Information Materials of Ministry of Education, School of Chemistry and Materials Science, Shanxi Normal University, Taiyuan 030031, China

**Keywords:** hydrogen evolution reaction, Pt nanoparticle, Co_3_O_4_ nanoarrays, electrocatalytic, synergistic effect

## Abstract

Electrocatalysts in alkaline electrocatalytic water splitting are required to efficiently produce hydrogen while posing a challenge to show excellent performances. Herein, we have successfully synthesized platinum nanoparticles incorporated in a Co_3_O_4_ nanostructure (denoted as Pt-Co_3_O_4_) that show superior HER activity and stability in alkaline solutions (the overpotentials of 37 mV to reach 10 mA cm^−2^). The outstanding electrocatalytic activity originates from synergistic effects between Pt and Co_3_O_4_ and increased electron conduction. Theoretical calculations show a significant decrease in the ΔG_H*_ of Co active sites and a remarkable increase in electron transport. Our work puts forward a special and simple synthesized way of adjusting the H* adsorption energy of an inert site for application in HER.

## 1. Introduction

Hydrogen is considered an ideal green energy carrier to build a carbon-neutral society in the future [1]. Electrocatalytic water splitting is an efficient and eco-friendly way of converting fluctuant wind and solar renewable power into high-purity hydrogen without carbon emissions [2]. The increasing renewable power production and hydrogen energy demands require alkaline water splitting with high-throughput production, which needs catalysts to show the best activities [3,4,5]. For hydrogen evolution reaction (HER), Pt-based electrocatalysts show outstanding catalytic performance for their moderate hydrogen adsorption/desorption energy (ΔG_H*_), whose high price and scarcity seriously hinder their lasting practical applications [6]. Therefore, nonprecious and earth-abundant materials such as transition metals have been explored as alternative electrocatalysts in recent years.

Among them, transition metal oxides (TMOs) show special physical and chemical properties [7,8]. In particular, spinel Co_3_O_4_ is of interest due to its low cost, excellent electrocatalytic properties, and high corrosion resistance in alkaline solutions [9,10,11,12]. When the reaction happens in alkaline media, the beginning step of the formation from H_3_O^+^ to H* is harder and slower for a lack of H^+^ in the electrolyte, which needs extra energy to break the H-O-H bond for water dissociation [13]. Thus, there is a useful way to boost overall hydrogen evolution rates by combining a component with the ability to accelerate water dissociation. According to reports, TMOs have been proven to facilitate water dissociation owing to their strong adsorption of OH^−^ intermediates [14,15,16]. And Fajín et al. [17]. showed by calculations that the water dissociation reaction will preferentially occur at corner or edge positions on the platinum particles that have low coordinated atoms on the surface. Interactions between the Pt nanoparticle and metal oxide supports lead to a rearrangement of electrons in both materials, and several atomic layers at the interface between the particles and the supports significantly affect the redistribution of electrons [18,19]. Also, it has been reported that the electronic metal-support interaction between Pt and Co_3_O_4_ is stronger than other TMO-based supports. For example, Jana et al. [20]. reported that Pt/Co_3_O_4_ synthesized by a low-temperature aqueous-phase process exhibits excellent cycling stability and achieves current densities of 10 mA cm^−2^ at overpotentials of 70 mV. Further, Gu et al. [21]. grew ultrafine Pt nanoparticles in situ on Co_3_O_4_ nanosheets, which exhibited excellent HER activity with an overpotential of only 34 mV at a current density of 10 mA cm^−2^. However, there is no detailed study on the mechanism of performance enhancement.

Apart from the selected chemical compositions of phases and the exquisite construction of the lattice interface, controlling the morphology of catalysts is also a rational and promising route for improving electrochemical activities, which stems from exposing more active sites through a larger specific surface area [22,23,24,25]. In addition, the electrical conductivity of materials acts as a critical factor in catalytic performance, which determines the transport rate of the electron to activate sites [26,27]. Metal-organic frameworks (MOFs) have attracted extensive research attention for their high specific surface area and tunable porosity and are favorable precursors for the construction of nanostructured TMOs [28,29,30]. Therefore, it will be a useful strategy to design excellent alkaline HER activity based on the above aspects. Some recent developments have led to a revival of TMO-based materials as HER electrocatalysts. But there is a need to explore hybrid materials based on TMO-based hybrid materials to achieve efficient HER.

Herein, we designed and prepared Pt nanoparticle-modified Co_3_O_4_ nanoarrays by electrodeposition and subsequent annealing treatments. The synergistic effect of Pt and Co_3_O_4_ leads to high HER activity of 37 mV to reach 10 mA cm^−2^ in alkaline solution. DFT calculations revealed that the interactions between Pt nanoparticles and Co_3_O_4_ supports lead to electron transfer from the Co_3_O_4_ interface to Pt. The electronic structure of Co_3_O_4_ and Pt nanoparticles was altered, resulting in the optimization of the H* adsorption energy of the surrounding low-activity Co sites while introducing Pt sites that can efficiently enhance electron transport, reduce ΔG_H*_, and then improve the electrocatalytic performance.

## 2. Experimental Section

The carbon cloth was repeatedly washed and dried with acetone, deionized water, and ethanol, and then stored for use. Solution A was prepared by dissolving 1.414 g of dimethylimidazole in 40 mL of deionized water, and solution B was prepared by dissolving 2 mmol of Co(NO_3_)_2_·6H_2_O in 40 mL of deionized water, followed by slowly pouring solution A into solution B, and the prepared carbon cloth (2 × 2 cm^2^) was immersed in the solution for 4 h at 40 °C, then removed and washed repeatedly with deionized water and ethanol, The resulting Co-MOF nanosheets were heated and insulated in a muffle furnace at 350 °C for 3 h with a rapid 2 °C/min to obtain the original Co_3_O_4_ nanosheets, named Co_3_O_4_.

Pt-Co_3_O_4_ is synthesized by electrochemical deposition. In the three-electrode system, Co_3_O_4_ is used as the working electrode, a graphite rod as the counter electrode, and a Hg/HgO electrode as the reference electrode. The cyclic voltammetry (CV) activation of the Co_3_O_4_ was carried out at a scan rate of 10 mV s^−1^ between 0.05 and −0.50 V with respect to the reversible hydrogen electrode (RHE) by cycling the Co_3_O_4_ for 20 cycles in 1 M KOH. The system was then transferred to 1 M KOH containing 0.15 mmol L^−1^ H_2_PtCl_6_ in 1 M KOH solution for further electrodeposition of Pt. Electrodeposition of Pt-Co_3_O_4_ is 0.05 to −0.50 V with respect to RHE for 20 cycles at a scan rate of 5 mV s^−1^. The sample was obtained by annealing at 450 °C in Ar/H_2_ (5% H_2_) protection for 2 h. In the same procedure, the Pt-CC samples were obtained directly with a carbon cloth as the working electrode. And 1 M KOH containing different concentrations (0.05 mmol L^−1^, 0.10 mmol L^−1^, and 0.20 mmol L^−1^) of H_2_PtCl_6_ were prepared.

## 3. Material Characterization

The surface morphology of the samples was characterized using the SEM (Merlin Compact, Carl Zeiss AG, Oberkochen, Germany), To further investigate the crystal structure of the samples, TEM (Tecnai G2 F30, Thermo Fisher Scientific, Waltham, MA, USA) and XRD (D8 Advance, Bruker, Billerica, MA, USA) were used, and XPS (Thermo Fisher, Waltham, MA, USA) was utilized for samples to characterize the surface chemical state of the materials.

## 4. Electrochemical Test

All electrochemical performances were measured at the electrochemical workstation (CHI 760E, Shanghai Chenhua Instrument Co., Shanghai, China). The electrochemical tests were carried out using a three-electrode system, with a carbon rod as the counter electrode, Hg/HgO as the reference electrode, the prepared catalyst as the working electrode, and the electrolyte as a 1.0 M KOH solution. Electrochemical performance measurement methods mainly include linear voltammetry curves (LSV, at a scan rate of 2 mV/s), cyclic voltammetry scans (CV, from 10 mV/s to 50 mV/s), AC impedance tests (EIS, in the frequency range of 0.1 to 10^5^ Hz), chronopotentiometry, etc. All voltage values were 95% iR compensated and converted to standard hydrogen electrode potential.

## 5. Results and Discussion

**Figure 1:**
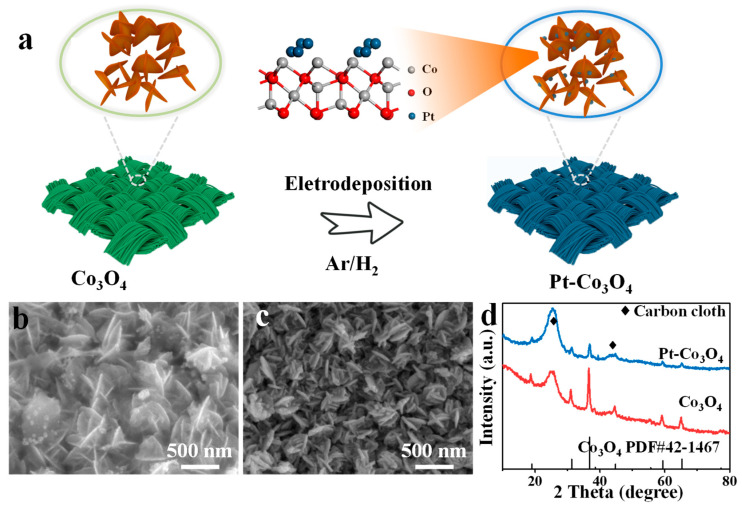
(**a**) Schematic diagram of the synthesis of Pt-Co_3_O_4_. SEM images of (**b**) Co_3_O_4_ and (**c**) Pt-Co_3_O_4_. (**d**) XRD patterns of Co_3_O_4_ and Pt-Co_3_O_4_.

Pt nanoparticle-modified Co_3_O_4_ arrays were obtained by the method shown in Figure 1a. Pt nanoparticle modified Co_3_O_4_ nanoarrays (Pt-Co_3_O_4_) were synthesized using the hydrothermal method, electrodeposition method, and annealing treatment in a tube furnace. The Co-MOF precursor arrays were first synthesized using the hydrothermal method using carbon cloth as substrate, followed by annealing treatment in air at 350 °C to obtain Co_3_O_4_ nanoarrays, and finally, Pt nanoparticle modification was carried out by the electrodeposition method as well as tube furnace annealing treatment in an Ar/H_2_ atmosphere (5% H_2_) to obtain Pt-Co_3_O_4_. The morphologies of samples are studied by SEM. Figure 1b,c show the SEM images of Co_3_O_4_ and Pt-Co_3_O_4_ samples. It can be seen that the Co_3_O_4_ arrays are uniformly grown on the surface of the carbon cloth, and the lamellar arrays are about 500 nm long and ~30 nm thick, while the Pt-Co_3_O_4_ obtained after modification by Pt nanoparticles still retained the original morphology. In addition, the optimized electrocatalytic performance for Pt content is shown in Figure S2. In order to analyze the structural information of the obtained samples, the crystal phases of the samples are further determined by XRD, and the results are shown in Figure 1d. For the XRD result of Pt-Co_3_O_4_, two typical broad diffraction peaks sited at 26.6° and 43.4° are clearly observed, which corresponded to carbon peaks of carbon cloth. It can be seen that the phase corresponds to the cubic Co_3_O_4_ phase (PDF#42-1467), and after the modification of Pt nanoparticles, the phase is still maintained, but the peak intensity is reduced due to the partial reduction of the Co_3_O_4_ surface during electrodeposition.

**Figure 2:**
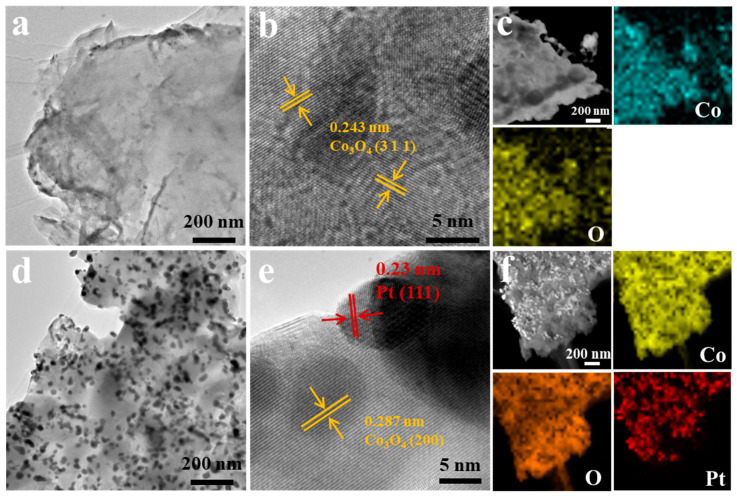
(**a**,**b**) TEM image and HRTEM image of Co_3_O_4_. (**c**) TEM mapping of Co_3_O_4_. (**d**,**e**) TEM image and HRTEM image of Pt-Co_3_O_4_. (**f**) TEM mapping of Pt-Co_3_O_4_.

Moreover, TEM is used to characterize the structure and elemental distribution of catalysts. As shown in Figure 2, the Pt-Co_3_O_4_ nanosheet structure exhibits an edge size of ~500 nm, which is consistent with the SEM analysis of the morphology. The average size of platinum nanoparticles was 15.35 nm, and their size distribution was determined based on TEM data as shown in Figure S3. Furthermore, high-resolution transmission electron microscopy results show that the obtained Pt-Co_3_O_4_ nanosheets have a crystalline facet spacing of ~0.243 nm, corresponding to the (311) crystalline plane of Co_3_O_4_, and a crystalline facet spacing of ~0.23 nm, corresponding to the (111) crystalline plane of Pt, indicating the successful introduction of Pt elements, which can bring abundant active adsorption and desorption for intermediate active species sites, which is conducive to promoting the electrocatalytic reaction. Element mapping also confirms the uniform distribution of O, Pt, and Co elements. In order to further confirm the content of Pt in Pt-Co_3_O_4_, the ICP analysis was used to investigate and verify that the content of Pt in Pt-Co_3_O_4_ is about 7.5 wt%.

**Figure 3:**
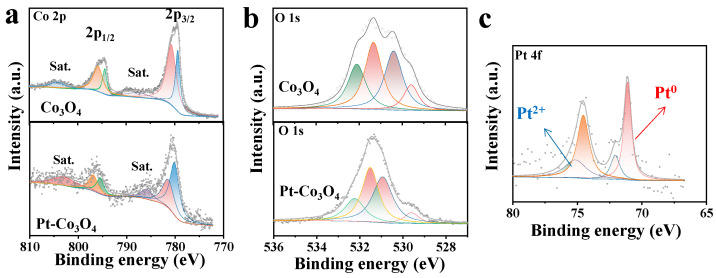
High-resolution elemental XPS spectra of Co_3_O_4_ and Pt-Co_3_O_4_: (**a**) Co 2p, (**b**) O 1s, and (**c**) Pt 4f.

To further study the elemental chemical state of the samples, XPS analysis was employed, as shown in Figure 3. The XPS spectra of Pt-Co_3_O_4_ confirm the presence of Co, O, and Pt elements on the electrode surface. The 2p orbital in elemental Co consists of two spin-orbit double peaks and two satellite peaks; the characteristic peaks at 793.7, 778.9, 798.6, and 781.3 eV correspond to Co^3+^ 2p_1/2_, Co^3+^ 2p_3/2_, Co^2+^ 2p_1/2_, and Co^2+^ 2p_3/2_, respectively, whereas the two satellite peaks were observed at 803.0 eV and 787.0 eV [31]. For the O 1s energy spectrum, four main peaks can be identified: the peak at around 531.2 eV is attributed to lattice oxygen, the peak observed at about 531.7 eV is related to oxygen vacancies, and the peak at around 532.6 eV is attributed to surface adsorbed water molecules [32]. For Pt-Co_3_O_4_, the peak observed near 529.7 eV is the M-O peak; the weakening of the bond is attributed to the formation of trace hydroxides by reduction of the Co_3_O_4_ surface during electrodeposition. In the spectrum of the Pt 4f orbital, it may be obvious to identify four main peaks. The peaks with binding energies of 72.1 eV and 74.7 eV correspond to metallic Pt, located at 72.9 eV and located at 76.2 eV, which can be attributed to Pt^2+^, confirming that Pt^4+^ is reduced [33,34]. And from XPS data, the platinum content was 8.49 a.t.%.

**Figure 4:**
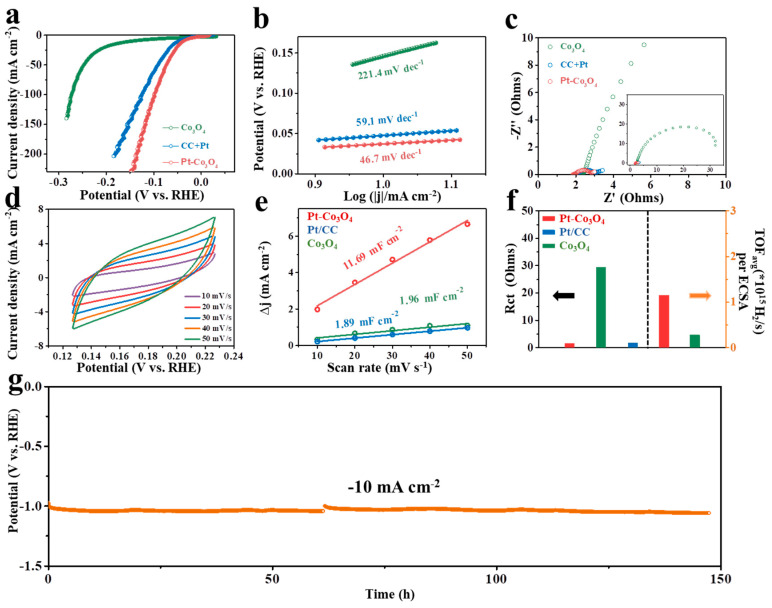
(**a**) LSV curves and (**b**) Tafel plots of the obtained catalysts for HER tests. (**c**) Nyquist plots of the obtained catalysts. (**d**) CV curves of D-Pt-Co_3_O_4_. (**e**) the calculated electrical double-layer capacitor (C_dl_) values. (**f**) TOFs at 100 mV and charge transfer resistance of the obtained catalysts. (**g**) Long-term running of Pt-Co_3_O_4_.

Further, the HER catalytic performance of as-synthesized electrocatalysts was also assessed in a 1.0 M KOH solution. Encouragingly, Pt-Co_3_O_4_ presented remarkable activity with an overpotential of 37 mV to reach 10 mA cm^−2^, which is lower than that of Co_3_O_4_ (376 mV) shown in Figure 4a. The performance improvement may be because of the insertion of carbon, which facilitates electron delivery. Figure 4b shows the corresponding Tafel plots, which are related to reaction kinetics. At the onset current density, the Tafel slope generated by Pt-Co_3_O_4_ (46.7 mV dec^−1^) was lower than that of Co_3_O_4_ (221.4 mV dec^−1^), indicating that Pt-Co_3_O_4_ in alkaline medium has a more efficient process. In addition, electrochemical impedance spectroscopy (EIS) was conducted to further evaluate the electrode reaction kinetics of catalysts. The Nyquist plots were fitted by the corresponding equivalent circuit model in Figure 4c, which illustrates the maximum conductivity of Pt-Co_3_O_4_ consistent with the fast HER kinetics.

Moreover, the electrochemical surface area (ECSA) of the samples is used to estimate the number of active sites exposed by electrocatalysts (Figure S4 and Figure 4d), which can be evaluated by calculating the C_dl_ from the CV curve [35]. Figure 4e showed that Pt-Co_3_O_4_ presented a considerably bigger C_dl_ (11.69 mF cm^−2^) than Co_3_O_4_ (1.96 mF cm^−2^), leading to much more active sites exposed in the HER test. The higher ECSA of Pt-Co_3_O_4_ might be attributed to the special hollow structure of MOF-derived. Importantly, under an overpotential of 100 mV, Pt-Co_3_O_4_ has a turnover frequency (TOF) of ≈1.12 (Figure 4f), suggesting that the number of active sites is greatly increased [36,37]. Besides, catalytic stability is also one of the virtual parts of evaluating the performance of catalysts. The long-term chronopotentiometry test was applied to examine the stability of Pt-Co_3_O_4_, as shown in Figure 4g. The initial current density of 10 mA cm^−2^ of Pt-Co_3_O_4_ displayed negligible degradation, respectively, after a 72 h electrocatalytic HER test, which confirmed the robust long-term catalytic stability in alkaline solutions. The above data implied that the fast electron transfer and abundant active sites modulated by the incorporation of Pt and a special hollow spherical structure could efficiently promote the hydrogen evolution reaction.

**Figure 5:**
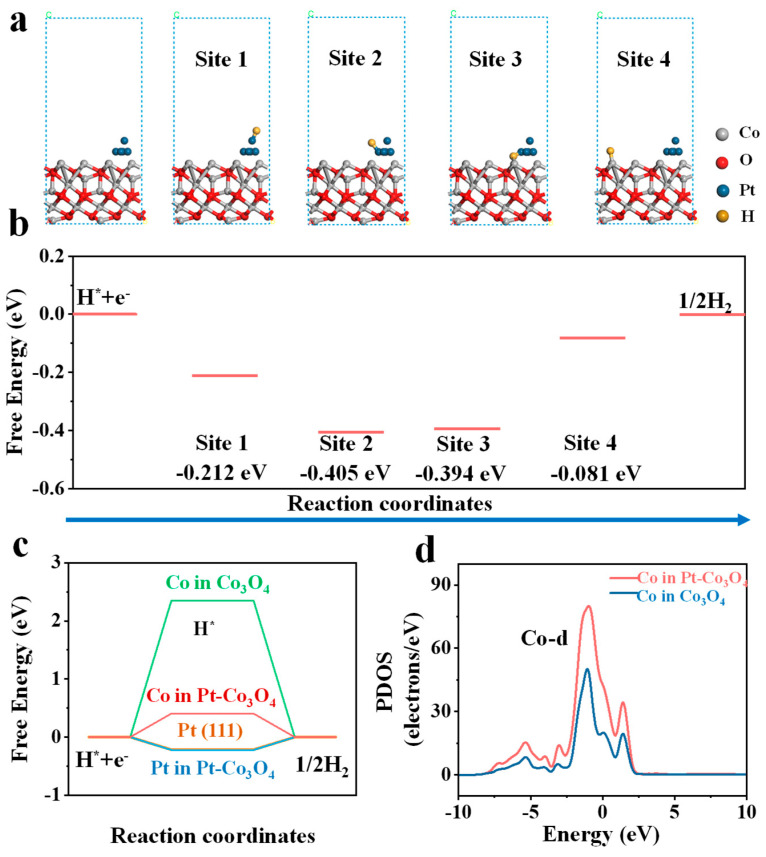
DFT calculation for HER performance. (**a**) The atomic models of Pt-Co_3_O_4_. (**b**) Gibbs free energy (ΔG_H*_) for HER at different sites of Pt-Co_3_O_4_. (**c**) The Gibbs free energy values (ΔG_H*_) for HER at different sites. (**d**) The partial density of states (PDOS) of the Co-d orbital.

To obtain deeper insight into the composite effects of Pt-Co_3_O_4_ on HER performance, Pt-Co_3_O_4_ was constructed for theory calculation using DFT for the improvement of catalytic activity, as shown in Figure 5 and Figure S5. The Pt site in Pt-Co_3_O_4_ shows the lowest adsorption energy (−0.212 eV). This value is close to the adsorption energy of the Pt (111) crystal plane (−0.174 eV), which shows a strong hydrogen adsorption capacity, suggesting that the Pt site is one of the main active sites in the catalytic process. Furthermore, as shown in Figure 5b, the results of ΔG_H*_ for different Pt sites and Co sites show that in Pt-Co_3_O_4_, the H* at the top of Pt (site 1, −0.212 eV) is close to the above case as these Pt sites are away from Pt-Co_3_O_4_. In addition, ΔG_H*_ is −0.405 eV on the Pt at site 2, indicating increased hydrogen adsorption at this site. Stronger hydrogen adsorption leads to a faster supply of protons to the reaction, but this also leads to a slow release of hydrogen at the active site, which can limit the overall HER activity. This is also consistent with the experimental results above, and the performance of Pt seems to decrease instead of increasing its content. The ΔG_H*_ at the interface (site 3) is −0.394 eV, indicating a gradual relaxation of hydrogen adsorption, and the ΔG_H*_ at the Co site at site 4 is −0.081 eV (the ΔG_H*_ of Co sites in Co_3_O_4_ is 2.342 eV, see Figure 5c), and some Co sites start to become new active sites, which is due to the fact that the interactions between Pt nanoparticles and Co_3_O_4_ supports lead to electron transfer from the Co_3_O_4_ interface to Pt (Figure S6). The electronic structure of Co_3_O_4_ and Pt nanoparticles was altered, and Pt nanoparticles activated some of the Co sites, which caused excess hydrogen adsorption at the interface and promoted hydrogen overflow from the surface of Pt-Co_3_O_4_ to Co sites, resulting in efficient HER activity [38]. Also, the PDOS in Figure 5d near the Fermi energy level of Pt-Co_3_O_4_ is stronger than that of Co_3_O_4_, indicating a higher carrier concentration, suggesting that the Pt-Co_3_O_4_ catalyst has a faster charge transfer kinetic rate.

## 6. Conclusions

In this study, we synthesized Pt nanoparticle-modified Co_3_O_4_ by a simple strategy, which presents a large surface area and provides high-efficiency electron conduction. Pt-Co_3_O_4_ exhibits low overpotentials of 37 mV to deliver 10 mA cm^−2^ in an alkaline solution. The experimental and theoretic simulation results show that the interaction between Pt nanoparticles and Co_3_O_4_ support led to electron transfer from the Co_3_O_4_ interface to Pt, which optimized the H* adsorption energy of the low-activity Co sites around the Pt nanoparticles, and the synergistic effect of Co and Pt sites reduced the ∆G_H*_ in the HER process, which in turn improved the electrocatalytic performance. Our work opens the door to practical optimization of the H* adsorption energy of low-active sites on HER performance.

## Data Availability

Data is contained within the article and Supplementary Material.

## Supplementary Materials

The following supporting information can be downloaded at: https://www.mdpi.com/article/10.3390/nano14110898/s1, Figure S1. SEM images of Pt-CC; Figure S2. (a) LSV curves and (b) Tafel plots of the obtained catalysts for HER tests. (c) Nyquist plots of the samples after immersing with different Pt concentrations; Figure S3. TEM image image of Pt-Co3O4 and Pt nanoparticles size distribution; Figure S4. CV curves of (a) Co3O4 and (b) Pt-CC; Figure S5. The atomic models of Co3O4 with Pt nanoparticles; Figure S6. Computational models and localized electric field distributions of Pt-Co_3_O_4_. Refs [39,40] are cited in Supplementary Materials.
