# Supplementary material for: Rational Construction of Pt Incorporated Co3O4 as High-Performance Electrocatalyst for Hydrogen Evolution Reaction
Source: Nanomaterials (Basel). 2024 May 21;14(11):898. doi: 10.3390/nano14110898 (PMC11173378; doi:10.3390/nano14110898)
Supplement: Supplementary file 1 [file nanomaterials-14-00898-s001.zip › nanomaterials-3021245-supplementary.pdf]

Support information for:

Rational construction of Pt incorporated Co_3O_4 as high-performance electrocatalyst for hydrogen evolution reaction

Peijia Wang[a], Yaotian Yan[a], Bin Qin[b], Xiaohang Zheng[a*], Wei Cai[a], Junlei Qi[a*]

[a]School of Materials Science and Engineering, Harbin Institute of Technology, Harbin, 150001, China.

[b]Key Laboratory of Magnetic Molecules and Magnetic Information Materials of Ministry of Education & School of Chemistry and Materials Science, Shanxi Normal University, Taiyuan, 030031, China.

*Corresponding author:

Name: Xiaohang Zheng, Junlei Qi

E-mail: zhengxiaohang@hit.edu.cn, jlqi@hit.edu.cn

Theoretical calculations

The calculations use spin-polarised density functional theory (DFT) in the general gradient approximation parameterised by Perdew, Burke and Ernzerhof[1]. In the geometric optimization, the convergence criteria for the force and energy variations are 0.1 eV $Å^{-1}$ and 2 × 10^{-5} eV $atom^{-1}$, respectively, and the self-consistent convergence criterion is 10^{-5} eV $atom^{-1}$. A plane-wave energy cutoff is set at 480 eV and the vacuum layer at 15 Å to avoid anthropogenic effects. A Hubbard correction (GGA+U) was applied to solve the problem of partial cancellation of the GGA self-interaction. The value of U for cobalt is 3.52 eV. The calculation of ΔG_{H*} assumes that the entire HER pathway consists of two steps: (1) adsorption of hydrogen on the catalytic site (*) from the initial state ($H^+ + e^- + *$); (2) releasing the product hydrogen ($1/2H_2$). The total energies of $H^+ + e^-$ and $1/2H_2$ are equal. Therefore, the Gibbs free energy of the adsorption of the intermediate hydrogen on a catalyst (ΔG_{H*}) is the key descriptor of the HER activity of the catalyst. The ΔG_{H*} is obtained by the equation：

$\Delta G_{H*} = \Delta E_{H*} + \Delta ZPE - T\Delta S$,

where ΔE_{H*}, ΔZPE and ΔS are the binding energy, zero-point energy change, and entropy change of H* adsorption, respectively. Herein, according to previous research by Norskov *et al.*[2], the value of $\Delta ZPE - T\Delta S$ is about 0.24 eV (>0). The calculation of ΔE_{H*} follows the following equation:

$\Delta E_{H*} = E_{surf} + H* - E_{surf} - 1/2\ E_{H2}$

where the E_{surf} + H* is the total energy of the system with one adsorbed H atom in a

supercell. E_{surf} and E_{H2} represent the energy of bare surface and H_2 gas molecules, respectively.

Calculation of TOFs

The TOFs of each electrocatalyst are calculated as follows:

TOF_{H2} = |J| * mA / ECSA * 1 C s^{-1} / 1000 mA * 1 mol e^- / 96495.3 C * 1 mol H_2 / 2 mol e^- * 6.022 * 10^{23} H_2 molecules / 1 mol H_2

= |J| / ECSA * 3.12 * 10^{15} H_2 s^{-1} per mA/cm^2

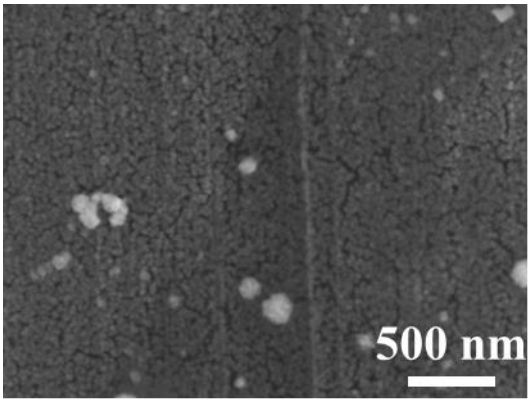

Fig.S1 SEM images of Pt-CC.

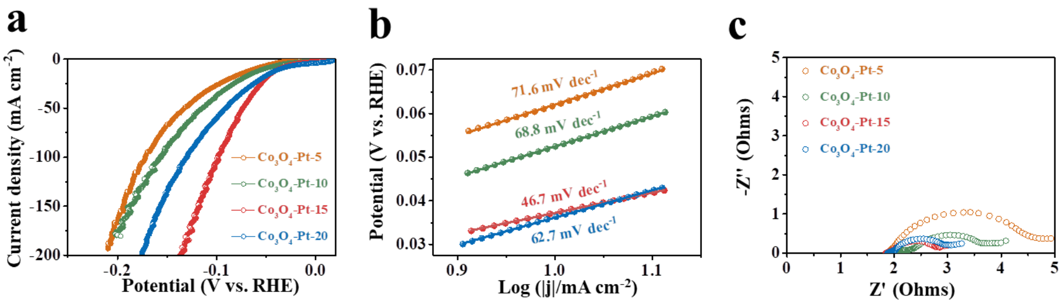

Fig.S2 (a) LSV curves and (b) Tafel plots of the obtained catalysts for HER tests. (c) Nyquist plots of the samples after immersing with different Pt concentrations.

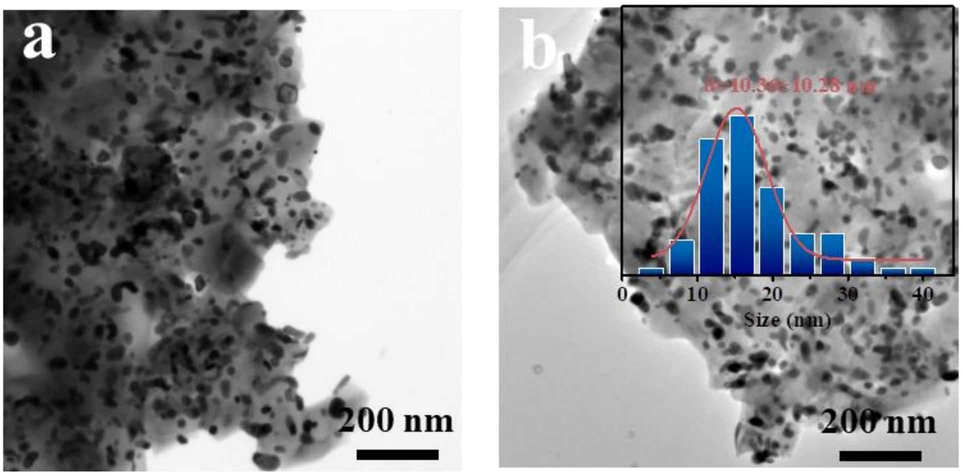

Fig. S3 TEM image image of Pt-Co_3O_4 and Pt nanoparticles size distribution.

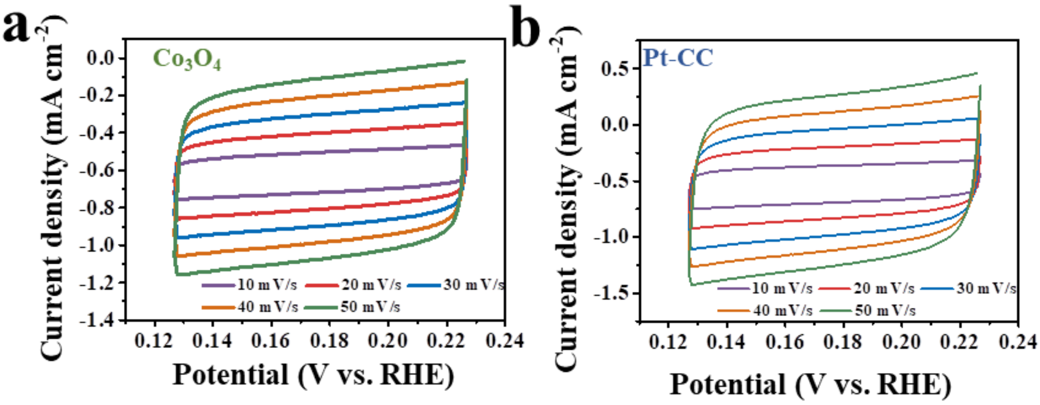

Fig. S4 CV curves of (a) Co_3O_4 and (b) Pt-CC.

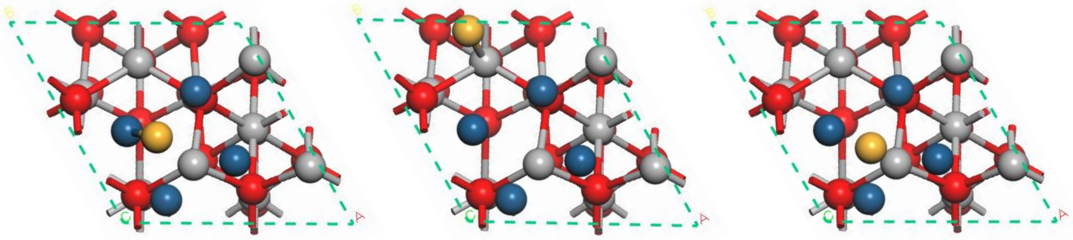

Fig. S5 The atomic models of Co_3O_4 with Pt nanoparticles.

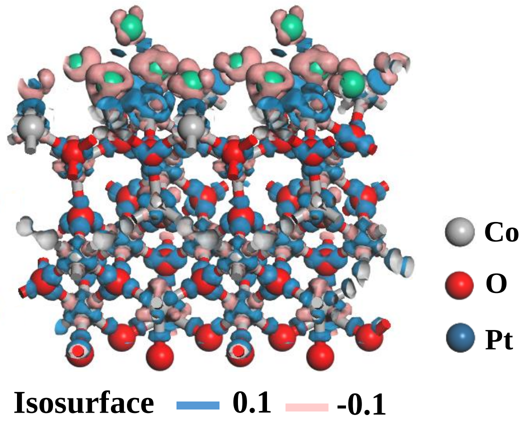

Fig. S6 Computational models and localized electric field distributions of Pt-Co_3O_4.

[1] J. P. Perdew, K. Burke, M. Ernzerhof, *Phys. Rev. Lett.* **1996**, *77*, 3865.

[2] J. K. Norskov, T. Bligaard, A. Logadottir, J. R. Kitchin, J. G. Chen, S. Pandelov, J. K. Norskov, *J. Electrochem. Soc.* **2005**, *152*, J23.
